# 

**Published:** 1893-08

**Authors:** 


					﻿By WILLIAM H. PALMER, M. D.
10 cts. a copy.
AUGUST, 1893.
$1.00 per year.
HALES*
JOVRNALtf
HEALTH
ESTABLISHED 18^4
l/QL XL
We.- 8.
OFFICE. 23 PARK ROW.
Entered as Second-Class Matter at the Post-Office, New York.
				

